# M. Sabatier on the Laws of Revulsion

**Published:** 1833-01-01

**Authors:** 


**Les Lois de la Révulsion.** Par *J. C. Sabatier*, M.D. Octavo, pp. 146. Baillière, 1832.

To this Essay was awarded the prize of the Society of Practical Medicine at Paris. The question proposed was in the following terms:—"What are the laws of revulsion? what is the assistance which the healing art may receive from it? and what the advantages and inconveniences of revulsive remedies, according to the cases in which they are employed, and the period of the maladies at which they are used?"

In a few preliminary observations, the author alludes to the distinction which was anciently made between the phenomena of derivation and of revulsion; the former epithet being used when the counter-irritation was produced near to the part affected with disease, and the latter when it was more remotely applied. The Greek authors admitted four kinds of revulsion: 1, from the superior to the inferior parts of the body; 2, from the right side to the left; 3, from the anterior to the posterior parts; and, lastly, from the internal to the external parts. It is well known that Hippocrates invariably let blood from a part as near to the seat of disease as possible; and this practice was followed, till the Arabian physicians introduced the very opposite fashion of revulsive bleeding. The above distinctions are now no longer regarded; and, in the present day, the epithets are often used synonymously, as it is very obvious that there is no essential difference of meaning, bleeding being designated sometimes as revulsive—at other times as derivative. It has been proposed, however, to limit the former appellation to the action which counter-irritants produce on the skin, such as that of blisters, rubefacients, &c.; and the latter to the effect of medicines on internal organs, as is exemplified in vomiting, purging, &c. Dr. Sabatier employs the term "revulsion" alone, and thus defines its meaning:—"It is that vital phenomenon, in virtue of which a morbid affection of any part is either gradually lessened, or quickly removed, by a certain action being established artificially, or naturally, in one or more other organs, situated at a greater or less distance from it;" and then he indulges in a long, uninteresting, and uninstructive disquisition on the properties of living bodies, and bewilders both himself and his readers by interpreting the meanings of such words as excitability, excitation, sensibility, and so forth; giving, what appears to us most unwise, the preference to the term "sensibility" to denote that property of living animal matter, whereby its power and qualities are liable to be changed and modified from without or within; take, for example, the skin—it may become either more or less vascular, more or less hot, more or less susceptible of pain, more or less exhalant. The appellation of excitability or mobility would be certainly more correctly expressive. The author then endeavours to explain the perplexing term "irritation:" he tells us that it is always the immediate result of increased excitement in any part, and is accompanied with an afflux and accumulation of the fluids in the capillary vessels, which may gradually become so distended as to lose their contractility, and thus occasion a stagnation or stoppage of the minute circulation, giving rise to the state of inflammation. The mere increased flow of blood, which constitutes the state of irritation, is frequently a phenomenon purely physiological and consistent with health, as is exemplified in the erection of the penis, clitoris, and mamilla. On the other hand, the specific characters of inflammation are, 1, the extreme distention of the capillary vessels; 2, the swelling; 3, the tendency to disorganization of the parietes of the blood-vessels, the pain, formation of pus, or destruction of the vital powers of the affected part. When we talk of irritation as a condition of disease, we allude to that state of a part which exists between the transition from mere increased vascular action to absolute inflammation. In considering the subject of revulsion, we shall find that the means employed for this end, in some cases, produce simple excitement—in others, complete irritation, and, in a third set, inflammation and its consequences. These means may be thus classified into two divisions; as they are applied to the exterior surface of the body, and as they are exhibited inwardly; and these again our author subdivides, according to the intensity or severity of their action: thus, sinapisms, stimulating embrocations, local hot-baths, dry cupping, &c. induce more or less powerful irritation—the actual and potential cauteries, the moxa, blisters, and setons, cause much deeper irritation, and even considerable inflammation. On the other hand, the application of leeches and the scarificators is followed by an irritative excitement, with a certain congestion of the capillaries, and a discharge of blood; while the effects of electricity and galvanism are to merely increase the excitability or mobility of the parts to which they are applied.

The internal revulsive remedies may be similarly grouped, according to the degree of their action, into excitatives, such as diuretics, laxatives; and into irritatives, as drastics, emetics, &c.; and inflammatory, or phlogistics, as acrid injections and virulent drugs, such as mercury, antimony, &c.

We have already mentioned that Nature sometimes avails herself of revulsive modes of cure, as when she induces certain acute diseases upon an already-existing malady: thus, we observe that erysiselas of the face disperses vesicular, pustular, squamous, &c. affections of the skin in other parts, and accelerates the resolution of some swellings, and the cicatrization of ulcers situated at a distance—that an acute cutaneous disease often checks diarrhœa and bronchitis; and, on the other hand, that inflammation of the stomach and bowels causes any rash or eruption to disappear—that a purging will arrest a catarrh, and gonorrhœal ophthalmia an attack of urethritis—that inflammation of the breasts will relieve hysteritis, and cynanche parotidea a hernia humoralis. To a certain extent, we can imitate many of the workings of Nature, as when we give sudorifics to stop a diarrhœa, or a drastic purge to cure an exanthema. But we return to the subject of external revulsives, and shall now adduce a few examples where the body, under the influence of disease, has resisted the ordinary operation of stimulating and irritative applications.

*Case* 1. A patient suffered from excruciating neuralgia of the testicle and spermatic cord. The endermic use of morphia was ordered; and, to obtain a raw surface as quickly as possible, an iron, heated to 212° of Fahrenheit was firmly applied for some time to the skin of the perineum; but no effect could be produced, although the operation was tried several times.

*Case* 2. A young man was labouring under acute "ramollissement" of the brain; he retained (a circumstance rare in such cases) the power of moving his limbs, and also the sensibility of all the surface. Sinapisms, made very powerful and repeatedly applied, did not even redden the skin. The ointment of the tartras antimonii produced no effect; and a large blister on the scalp only a very trifling irritation.

*Case* 3. A patient had a very severe attack of erysipelas of the face. Mustard poultices and blisters were either quite inefficacious, or caused only a slight exhalation on the skin.

*Case* 4. A physician suffered a smart onset of acute rheumatism. In a few days a metastasis to the diaphragm occurred, threatening suffocation. The most powerful rubefacients quite failed in producing any counter irritation.

The same obstinate resistance to the ordinary effects of the remedies employed is occasionally observed in the internal exhibition of revulsive medicines. In colica pictonum and in paralysis, the most acrid drastics will sometimes utterly fail to purge; whatever very much exhausts the energies, and impairs the sensibility of the system, lessens in proportion its susceptibility of the action of revulsive agents: for example, a woman, weakened by a uterine hæmorrhage, was carried to the hospital, and in order to re-animate the circulation, strong sinapisms were applied to the calves of the legs for three hours; but no redness ever was produced. In the last stage of many diseases, which are characterized by great debility, the most potent irritants are of no avail; and, as a general conclusion, Dr. Sabatier announces that the local action of a revulsive remedy is the more energetic, and its immediate effects the more obvious, in proportion as the part to which it is applied is more healthy and normal. We frequently observe that, although a counter-irritant fails in producing any effect at the time at which it was applied, no sooner are the symptoms of the malady relieved, than the irritation of the surface shews itself; thus, in the case of the patient on whom the heated iron failed to vesicate the perineum, when the severe neuralgia gradually abated, the part appeared to have been cauterized. In the third case above narrated, from 10 to 14 days elapsed before any redness of the thighs and legs, on which the sinapisms had been laid, appeared; this was followed by numerous vesicles, severe excoriation, and even gangrene at several points of the skin. The same consequences occurred in cases fourth and fifth, after an interval of three and four days from the application of the irritants. Our readers will be satisfied with the detail of the facts, and have, no doubt, little wish to be indulged with the "whys" and "wherefores" of authors, upon the ænigmata of sympathies. Dr. Sabatier descants largely upon this topic, but we are not tempted to follow his example. His remarks on bloodletting, as a revulsive remedy, are pertinent and judicious, and place the question in a very fair point of view. He observes that, of late years, this most potent therapeutic agent has been almost entirely excluded from the domain of revulsion, although once it was regarded as by far the most important denizen: the cause of this mutability arises from the erroneous or ill-understood opinions of authors, on the precise meaning of the terms employed. If revulsion is admitted to take place, whenever any diseased organ is restored to a state of health, in consequence of a peculiar action being set up in another viscus or tissue, and in virtue of the sympathies between them, which either exist naturally or are produced by art, then we must regard bleeding as a revulsive remedy, since we know that it is capable of effecting such a result: thus, in a case of suppression of the catamenia, a plethora in the head often ensues—bleeding relieves such a state, and the discharge speedily is restored. The analysis of the effects of the remedy or modus operandi may be thus stated:—1. An evacuation of blood; 2. A diminution of the excitability of the system generally, and, consequently, of the brain; 3. Diminution and cessation of the cerebral plethora, and, 4, Restoration to a normal state of the circulation in the brain and in the womb; 5. Restoration of the healthy equilibrium in the functions of these organs; 6. Return of the catamenia. Such is the categorical announcement of the author, which, when tried by the logic of common sense, means that amenorrhœa, when depending on an overcharged condition of the vascular system, is best relieved by bloodletting, whether from the foot or arm it matters little; it is the simple effect of a known cause, and to this simple averment we should be contented to adhere. To call it a revulsive action, implies that the blood is diverted directly from the vessels of the brain to those of the womb, or perhaps rather to the vein which has been opened; but of this we have no proof, and we shall be more correct in simply stating, that less blood is now circulated through the uterine vessels than before the operation, and that it is entirely owing to this diminution that the discharge has returned. Now this, if true, is opposed to all our ideas of revulsion, the essential features of which is excitement or irritation of a part which is sound, to relieve one that is diseased. With regard to topical bloodletting by leeches, it is rather from the the depletion which they occasion, than from any derivative congestion of blood in the part to which they are applied, that their benefit chiefly arises; in some cases, however, especially where the bites are followed by considerable pain and redness, they are entitled to be classed among revulsives.

A very common error among medical men, is to confound those remedies which deserve this appellation with mere irritants or stimulants. A few examples will best illustrate how distinct they are in action and effects :—A blister to the chest in pneumonia operates as a revulsive ; a blister to the scalp in low typhus fever, sinapisms to the feet in exhaustion from hæmorrhage, or to the stomach, in poisoning from narcotics, act as stimulants. Similarly contrasted phenomena are often induced by Nature ; a chronic eczema on the leg has been cured by an attack of erysipelas on the part ; scrofulous ulcers on the face and neck, many cutaneous diseases, and different sorts of tubercles, enlarged glands, &c. have disappeared from the same cause, and well illustrate the agency of a natural topical stimulant : and, reversing the case, we find that erysipelas, when it has been the primary disease, is often best cured by a blister, applied directly on the inflamed surface, as recommended by Baron Larrey. It must be obvious that it would be quite incorrect to designate such as examples of revulsive treatment. Were we to enumerate all the diseases, in which bonâ fide counter-irritants are usefully employed, very few in the nosological code would be omitted ; in diseases of all structures and textures, in acute and chronic, in general and local maladies, they often are most powerful remedies ; it is only in such as are connected with, or are more immediately dependent upon, a change in the properties of the fluids, that their applicability or efficacy is not prominent and direct, as in scurvy, syphilis, &c. It is a matter of some importance, in the treatment of a case, to determine what are the situations to which revulsives can be applied with most benefit and relief, and we shall probably find it advantageous, in Dr. Sabatier's opinion, to adhere to the following rule.—At the commencement of an acute disease, in which some local symptom or symptoms predominate, they should be applied as far as possible from the seat of the malady ; on the contrary, when the disease has reached its acmé, and in all chronic cases, the nearer that they are applied, so much the better. Certain portions of the surface of the body appear to sympathise very closely with certain others—thus, the skin on the side of one thigh, or of one arm, has an especial sympathy with the corresponding portions on the opposite limbs ; and, from many observations of cutaneous pathology, it appears that the skin which borders on all parts covered with hair, has a particular consentaneous alliance, one portion with another, in a state of disease. An extended experience has led Dr. Sabatier to the following conclusions :—

1. In certain pulmonic diseases, revulsive remedies ought to be applied to the inner surface of the arm, or to the hams.
2. In cerebral diseases, to the soles of the feet or to the ancles.
3. In affections of the diaphragm, to the soles of the feet (the sympathy between these parts is well illustrated by the effects of tickling the soles.)
4. In diseases of the peritoneum, intestines, and womb, to the inner surface of the thighs.
5. In stomach complaints, to the epigastrium.
6. To the nape of the neck in many maladies of the eyes, and in such nervous diseases as depend on a chronic inflammatory state of the brain or of its membranes.
7. To the sinciput, in amaurosis and incipient cataract.
8. To the hypogastrium, in affections of the bladder.
9. To the mammæ in menorrhagia, and some other diseases of the womb.

Barther states that it is generally most advantageous to apply all revulsive agents on the same lateral half, right or left, of the body, as that in which the diseased part exists, as there is a most powerful mutual sympathy between all the organs situated on one side of the median line.

The author offers some sensible remarks on the employment of blisters in various diseases, and shews that we must not forget that they act as stimulants as well as revulsives, and, if injudiciously timed, will aggravate all feverish and inflammatory symptoms ; the febrile irritation must be sufficiently appeased beforehand, and the affected organ be previously brought to that point, at which the new exciting cause will not induce any renewal or increase of morbid activity, but will remove such as already exists in it.

Having pointed out the precautions which must be observed in the employment of revulsive means in acute diseases, he next proceeds to illustrate their efficacy in very many chronic cases, as in old affections of the joints and of the air-passages;—in hydrops pericardii (a seton;)—in obstinate ophthalmia, otorrhœa, rheumatism, disease of the spine;—in severe neuralgia;—in paralysis, epilepsy, enlargements and congestions of the abdominal viscera, &c. Even in confirmed phthisis and in cancer of the stomach, they have been used with advantage, if not with success. M. Bouchard relates the following very interesting case.

A young girl, whose mother had died of tubercular consumption, was brought to Dr. Biett; she had the phthisical look, and was much emaciated; and occasionally expectorated blood. A distinct pectoriloquism, and a well-marked gurgling noise, during cough and deep inspiration, were heard below the clavicles; a cautery was ordered to be applied over the spot where the pectoriloquism existed; extract of conium was given, and a milk diet recommended: in a few months she recovered her strength and health completely. In such cases it is proper to encourage a free discharge from the ulcerated surface, for a considerable length of time.

Mr. B. Bell, in his work on ulcers, gives a very prudent advice, to establish a seton in the neighbourhood of the wound, after extirpation of the scirrhous mamma.

There is another class of diseases in which revulsive remedies have been used with advantage, namely, in hypertrophies; by leeches and scarifications in the early stage, and afterwards by the formation of an issue, seton, or other drain. We must refer the curious reader to the memoir of Baron Larrey on this subject, recently published in his "Clinique Chirurgicale exercée dans les camps, et les hôpitaux," tome III, 1830.

When rheumatism is transferred from any joint to an internal organ, Dr. Sabatier says that revulsives applied on the part originally affected, are the most "heroic" means of cure which can be employed, for example, very strong and acrid sinapisms, blisters, and cupping. In retrocedent exanthemata their good effects are equally conspicuous; in such cases the most prompt and sure is a hot-bath, and this should always be had recourse to along with local applications. The following two cases we consider worthy of being detailed.

1. A young man had for two years suffered from severe colicy pains, which were accompanied with diarrhœa; the epigastrium was exceedingly tender, and especially after taking food; frequent vomitings and great emaciation; every sort of stomachic, anti-spasmodic, and soothing medicines had been fruitlessly tried. Dr. Bourdier, who was now consulted, ascertained that most of the symptoms followed soon after the healing of a large boil on the thigh; that they were generally mitigated whenever any small ulcers appeared on the toes, and aggravated when they were dried up; a blister was applied over the site of the boil, and sinapisms to the toes; and no sooner was a discharge established, than this patient began to recover, and speedily he was made quite well.

*Case* 2. A man had had for a long time an eruption on the fore-arm—this suddenly disappeared, and a discharge from the urethra came on. M. Petit, to whom he applied, at first thought that it was a case of gonorrhœa, but the man swore that he had not exposed himself to any contagion, and that his wife was free of all disease. On further questioning him, he told M. Petit, that he had been subject to the cutaneous affection; a blister was applied to the fore-arm; the "dartre" re-appeared, and the discharge immediately ceased.

Many spasmodic and nervous affections are best counteracted by revulsive agents ; thus, vomitings, which have resisted every other means, have yielded to sinapisms, or to moxas applied to the epigastrium ; also hiccup, which in some cases is so distressingly obstinate, has at once been relieved by a scalding hot fomentation ; and the same application is equally useful in cases of spasm affecting the neck of the urinary bladder.

In amenorrhœa, and many other maladies of the womb, no means afford so much relief as leeches applied to the upper part of the thighs, and to the vulva, and if the hæmorrhoidal flux has been checked, the anus is by far the most proper situation from which a topical bleeding can be promoted. In women who have reached the critical period of life, it is advisable that leeches be applied rather to the anus than to the vulva, in order, if possible, to act as a means of revulsion upon the circulation of the womb, which is then so liable to disease.

The following practical hint in the employment of the pediluvium, is worthy of attention.

When our object is to produce an afflux of blood to, and a temporary irritation of, the lower extremities, the bath should be made only moderately warm, and the temperature raised by gradual additions of boiling water, instead of immersing them in very hot water at first ; the revulsion is thus more certainly obtained, and the system, especially of irritable patients, not exposed to any dangerous excitement. Instances have been known, in which apoplectic patients have suddenly died, while their feet were plunged in very hot water, in which mustard powder had been mixed ; the impression on the nervous system, re-acting on the sanguiferous, had been too strong, and had aggravated the very condition which they had been intended to obviate.

We have discoursed so largely on the revulsive remedies which are applied externally, that we have not space to do more, than simply to allude to the more prominent operation of such as are exhibited inwardly. Emetics and purgatives are often singularly useful in cerebral affections, and injuries ; in amaurosis and ophthalmias ; in inflammations of the mouth and throat ; in croup, hooping-cough ; in most cutaneous diseases ; in dropsies of the different cavities and of the cellular tissue ; in many anomalous pains of the limbs and of the trunk, &c. Other mucous surfaces may be acted upon by appropriate stimulants, so as to produce a revulsive effect. A sudden stoppage of a gonorrhœa has been followed by a severe neuralgia over the tendo Achillis, and has been as suddenly removed when the discharge was brought back by an ammoniated injection ; the alternations of a urethral, and ophthalmic purulent flux are well known ; and obstinate headaches, noises in the ears, wandering pains in the limbs, and many nervous maladies have speedily vanished, when a suppressed otorrhœa returned.

Such is a general view of the contents of Dr. Sabatier's essay. We have deemed it a wiser plan to give an analytical, than a judicial, or criticising review of its pages, as our readers will thereby be enabled to form their own opinions of its merits. We cannot, however, congratulate the author on having executed his task in the most philosophic manner. There is too much theoretical disquisition and dispute, too few facts, and little or no attempt to draw general principles from such as are adduced. This is the only method by which we may hope to arrive at the knowledge of general laws, such as the laws of revulsion, or of any other operation or function of the living body ; for what else do we mean by the term "general law," but that a host of particular facts, or specialities agree in certain qualities or powers ; and how, therefore, shall we become acquainted, except by guess-work, with the latter, unless we have accumulated and arranged the former ? What our author ought to have done, is, to have amassed, and brought into a focus, every well established occurrence of revulsion, such as is observed in the re-establishment of a gonorrhœal flux to cure certain ophthalmias ; the use of drastics and hydragogues in water of the brain ; the formation of an issue in many chronic diseases, &c. and from these he ought then to have cautiously deduced the laws which are found to apply to them all. Our parting advice to Dr. Sabatier is "study the Baconian philosophy."

